# Relatively (im) material: mtDNA and genetic relatedness in law and policy

**DOI:** 10.1186/2195-7819-9-4

**Published:** 2013-05-28

**Authors:** Caroline Jones, Ingrid Holme

**Affiliations:** School of Law, University of Southampton, Southampton, UK; School of Medicine and Dentistry, Plymouth University, Peninsula, UK

## Abstract

Mitochondrial donation poses the latest regulatory challenge for policy-makers in the context of assisted conception. Since 2010 the Human Genetics Commission, the Human Fertilisation and Embryology Authority and the Nuffield Council on Bioethics have all considered the policy implications of permitting use of these techniques in treatment. The Nuffield Council on Bioethics reported its recommendations in June 2012 following a consultation on the ethical issues raised by these techniques; and a separate consultation by the Human Fertilisation and Embryology Authority in conjunction with Sciencewise-ERC followed in September 2012. Matters for consideration included the potential relationships created by the use of three parties’ genetic material and the associated ramifications, eg whether or not there is a need to establish records of such donations and, if so, to whom should information later be provided? Thus, mitochondrial donation poses both novel and familiar questions about the ‘genetic family’, ‘parentage’ and ‘identity’. This article explores some of the ways in which mitochondrial DNA is constructed as relatively (in) significant in recent Parliamentary debates, policy and consultation documents. It reflects on the ways in which the role of some genetic connections, or lack thereof, are mediated in law and policy.

## Introduction

The development of mitochondrial donation or replacement techniques poses the latest regulatory challenge for policy-makers in the context of assisted conception. Since 2010 three key UK policy-advisors/makers, notably the Human Genetics Commission ( 2010 ), HFEA ( 2011 ) and the Nuffield Council on Bioethics ( 2012a ) have considered the policy implications of permitting the use of these techniques in treatment cycles. The NCOB reported its findings on June 12, 2012 ( Nuffield Council on Bioethics 2012b ) and the HFEA, together with Sciencewise-ERC (http://www.sciencewise-erc.org.uk/), launched its public consultation on the ethical issues raised by these techniques in September 2012^a^. We use the phrase ‘mitochondrial donation’ throughout, as adopted by the NCOB, but note that the HFEA utilised the term ‘mitochondrial replacement’ in its 2012 consultation and associated documentation. Similarly, we refer to ‘mutated’, ‘faulty’ and ‘unhealthy’ mitochondria in the discussion below as these are commonly used terms in the literature, rather than us seeking to make a ‘judgement’ about the biological materials and processes *per se* (more detailed consideration of the nuanced distinctions regarding such terminology falls outside the scope of this paper).

Among the numerous ethical and legal questions raised by mitochondrial donation is the broader consideration of the (im)permissibility of ‘germline therapies’ *per se*, and whether or not they should be treated as a useful test case for ethical issues involved in future potential nuclear DNA/germline modification^b^. Within this context, both familiar and novel questions about the ‘genetic family’, ‘parentage’ and ‘identity’ can be found. The focus of this paper is the examination of some of the ways in which mitochondrial DNA (mtDNA) is constructed as relatively (in) significant in recent Parliamentary debates, policy and consultation documents, in order to reflect on the ways in which the role of genetic connections are mediated in policy formation. The paper explores both ‘parentage’ & ‘identity', and reflects on the framing of the debates, in order to consider whether there is anything novel about mitochondrial donation that causes the current paradigm(s) of understanding (in Anglo-Welsh law and policy) to fall apart.

Prior to embarking on the main body of the paper it is useful to pause briefly to consider why techniques of mitochondrial donation were developed, and what each method entails.

### Biology 101

Mitochondria are present in almost all cells in the human body (red blood cells are an exception), and produce the energy the body needs to function. Mitochondria contain a small amount of DNA (mtDNA), usually only inherited matrilineally through the mother’s eggs ( Schwartz & Vissing 2002 ). To give a sense of scale, mtDNA accounts for an estimated 0.1% of our DNA, and has 37 genes thought to govern mitochondrial function, 13 of which code for proteins; whereas nuclear DNA makes up an estimated 99.9% of our DNA, with circa 25,000 genes^c^. However, cells have a *population* of mitochondria and, depending on the cell type/function, may contain a few hundred or several thousand. Whilst many people have low levels of mutated mitochondria in their cells, with little or no difficulties ensuing, those with higher levels can have serious health issues. Inherited mitochondrial disorders have – at present – no known cures, and may have seriously disabling symptoms, with the potential to cause death in babies and young persons (although the severity of symptoms and prognosis inevitably varies between people). While some mitochondrial diseases are caused by mutations in *nuclear* DNA (nDNA) rather than in mtDNA, the current techniques - and therefore the policy developments in this area - are focused solely on the avoidance of the transmission of mutated mtDNA *per se*. Consideration of nDNA modification to ameliorate or prevent mitochondrial conditions accordingly falls outside the focus of this paper.

Currently, women who wish to have children and avoid passing on mtDNA conditions have few options. They can seek to use donated eggs, provided by a woman without a history of mitochondrial condition(s); apply for adoption, or seek an arrangement with a surrogate mother willing to use her own eggs or donated eggs. In order to *reduce* the risk of transmission, women can elect to have preimplantation genetic diagnosis (PGD) to estimate the levels of mutated mitochondria in a given embryo. However, the PGD route is not suitable for all conditions, especially those which – when severe – have fatal outcomes, as the levels of mutated mitochondria in early embryos are a poor predictor of the severity of the disorder. Nor can PGD predict if the embryo will develop into an individual with a high level of mutated mitochondria in *all* their tissue types, or only in some or one. Hence, PGD is not suitable for all women, including those with high levels of mutated mitochondria; leaving aside any issues pertaining to accessing these technologies. Finally, prenatal diagnosis (PND) can be utilised, but in addition to the limitations outlined above regarding PGD, women (and partners) may be faced with deciding whether or not to terminate their pregnancies, a (potentially) difficult decision exacerbated by the uncertainty in predicting mitochondrial conditions and their severity. Hence, the use of mitochondrial donation techniques would enable these women to have a genetically related child (through their nDNA), if they so wished, whilst simultaneously avoiding the possibility of transmission of ‘faulty’ mtDNA to future generations.

### Mitochondrial donation techniques and the policy timeline

Two techniques have emerged, ‘pronuclear transfer’ and ‘maternal spindle transfer’. Both would create children born with nDNA from their parents’ sperm and egg^d^ plus healthy mitochondria from an egg donor, unrelated to the mother; hence, giving rise to a child with genetic connections to three people. The HFEA and NCOB have provided accessible explanations and diagrams of both methods which we will not repeat here^e^. The key distinction is that pronuclear transfer (PNT) involves the creation and use of embryos, or more accurately 1 day old zygotes (one of which is discarded), whereas maternal spindle transfer (MST) uses eggs (one of which is discarded). Hence there are at least three, and potentially four individuals providing gametes in each cycle, depending on which technique is used.

Both methods are currently being developed under a research licence, granted by the HFEA, but are not yet lawful for use in treatment cycles^f^. The Newcastle University group leading this research was initially granted a licence in 2005^g^ and some success regarding PNT was reported in 2008^h^, resulting in calls for the HFEA to be permitted to licence this technique for treatment in humans^i^. Around this time the first ‘three-parent baby’ media headlines emerged^j^ (see below). Ultimately, the Human Fertilisation and Embryology Act 2008 (HFE Act 2008) amended the 1990 Act of the same name – resulting in the insertion of 3ZA(5) HFE Act 1990^k^. This section enables the Secretary of State to introduce Regulations to Parliament in the future that (subject to Parliament’s approval) would permit the use of eggs or embryos that have undergone techniques such as PNT or MST in treatment cycles for people seeking to avoid the transmission of ‘serious mitochondrial disease’^l^. In preparation for placing such Regulations before Parliament, in January 2011 the then Secretary of State for Health, Andrew Lansley, invited the HFEA to report on the ‘safety and efficacy’ of these techniques^m^ and in 2012, in conjunction with the Secretary of State for Business, Innovation and Skills, he invited the HFEA and Sciencewise-ERC to undertake public dialogue on these developments (http://www.hfea.gov.uk/6896.html). As outlined above, for our purposes in this paper, two key focal points have emerged: parentage and genetic contribution and the framing of potential implications for the child’s identity.

### Parentage: two, three or ‘fractional’?

For our purposes ‘parentage’ is taken to mean *genetic* parents^n^ leading to the question as to whether having genetic contributions from three distinct ‘providers’ has (or should have) any legal and/or social significance?^o^. References to ‘parents’ were evident in the relevant Parliamentary debates, with the polarity of views highlighted by two examples: Lord Alton claimed that ‘adoption would be better than creating three genetic parents’; whereas Lord Walton was clear that ‘the idea that the resulting child has three parents is a nonsense’^p^. In many respects, therefore, this is the *familiar* question about parentage and the (assumed) unity of meaning of the terms ‘parent’, ‘mother’ and ‘father’ (potentially) disrupted by, for example, surrogacy and donor conception^q^. But, is the addition of a third party’s *mitochondria* (and hence mtDNA) sufficient to bring about a shift in legal policy?

### Media headlines

A brief look at some of the UK media headlines on mitochondrial donation in 2008 and 2011 might point in this direction. These examples illustrate the media’s construction of the techniques in relation to ‘parents’ (emphasis added):

- A step towards *three*-*parent babies*? (Nature)^r^
- *Three*-*parent embryo* formed in lab. BBC News ( 2008 )
- *Three*-*parent babies* a step closer after watchdog gives research go-ahead despite ‘life meddling’ fears. (Daily Mail) ( Derbyshire 2011 )
- *‘Three-parent’* IVF babies on their way. (New Scientist blog) ( Hamzelou 2011 )
- *Three-parent* IVF, and baby makes 4. … [technique] that creates children with *one father and two biological mothers*. (Sunday Times)^s^
- *Babies with THREE parents* and free of genetic disease could soon be born using controversial IVF technique. (Daily Mail)^t^
- Scientists seek to implant embryos with *genetic material from three parents*. (The Guardian) ( Boseley 2011 )

The Sunday Times highlighted the novel aspects thus: ‘the new technique would *for the first time involve a third person contributing genetically*’; and went further, ‘raising the question of what rights, if any, such *“fractional parents”* would have in relation to the child’?^u^. Clearly, classification as (some kind of) ‘parent’ was the media’s starting point for understanding the contribution of the mitochondrial donor, with a need, in the latter example, to clarify their legal position *per* the child (but no indication of any consideration of the *child’s* status in relation to the adult)^v^. Interestingly, however, it is *not* the first time a third person could contribute genetically through the use of ARTs, as cytoplasmic donation/ooplasmic transfer attests ( Robertson 1999 ); but this technique was not licensed in the UK and has since been prohibited in some other jurisdictions^w^, with no reported follow-up studies^x^. Accordingly, there is little data available, and no robust conclusions that could be drawn upon in order to inform the current policy challenges (favourably or otherwise).

### Policy constructions

In 2010 the HGC expressed concern regarding the media headlines:

Despite newspaper headlines describing the possibility of ‘children with three genetic parents’ it is not clear that the *donor of the mitochondria* should in any way be regarded as a *progenitor* (or a *gamete donor* for the purposes of the framework legislation) *any more than a kidney or bone marrow donor should be regarded as progenitor of the recipient*. … it might be decisive that the mitotchondria do not participate in fertilisation, … [they] are replicated rather than procreated … down the generations.^y^ (emphasis added).

Similarly avoiding the loaded term ‘parent’, the HFEA’s Ethics and Law Advisory Committee^z^ asked the question of ‘how would the *mitochondria provider* be classified?’ (emphasis added), noting that some people may view the introduction of a third person’s genetic material as less ‘novel’ due to their familiarity with bone marrow transplants^aa^. Finally, the NCOB, in its call for evidence, enquired in the following language:

After the use of these techniques, children would inherit nuclear DNA (around 25,000 genes) from their *parents*, and mtDNA (13 genes) from the *donor of the egg*. What might the use of these techniques signify for the relationships of the resulting child to the *three adults* with whom it shares a genetic connection?

The HFEA and the NCOB both raise the question as to the appropriate categorisation of the mitochondrial/egg donor, which we return to below under ‘practical matters’. However, on the issue of one person having genetic contributions from three parties, as highlighted by the HFEA re bone marrow transplants, the NCOB is clear that whilst this does involve the incorporation of a third person’s genes into patients’ bodies, ‘these changes are not inherited by the patients’ children’^bb^ and therefore may be distinguishable. Not only would mitochondrial donation result in a permanent modification of the child’s germline, but if female - given the matrilineal transmission of mtDNA - she would pass on the ‘acquired’ mtDNA to her future offspring/generations^cc^.

### Law

We are familiar with the separation of genetic and gestational motherhood following IVF, egg donation and surrogacy; but would a separation into *major and minor genetic contributors* create any difficulties regarding the legal constructions of mothers? This seems very unlikely, given the primacy accorded to *gestation* in Anglo-Welsh Law^dd^; and while the HFE Act 2008 can countenance two *female parents,* it cannot countenance *two mothers* – where two women are recognised together as legal parents they are done so as ‘mother’ and ‘parent’ respectively^ee^. If mitochondria were passed patrilineally it would have been interesting to see if mitochondrial donation had any impact on, for example, child support provisions, given the usual focus on paternal genetic ties.

Other jurisdictions have been more open to recognising three legal parents. For example, in 2005 the New Zealand Law Commission^ff^ tabled the possibility of recognising three legal parents where donor insemination was used; and in 2007 the Court of Appeal in Ontario, Canada, made a declaration of parentage in favour of a lesbian co-mother as the child’s third legal parent (where DI was used)^gg^. These instances illustrate the flexibility that *may* be exercised in some jurisdictions, even where there were ‘only’ two genetic contributors but three persons involved in parenting the child. However, it does not seem likely that the provision of mtDNA *per se* will disrupt the current paradigm regarding ‘parentage’ in Anglo-Welsh Law. Despite this apparent clarity, and as we have already noted, the matter of how mitochondrial donors are to be classified does have tangible policy outcomes that must be addressed.

### Identity: ‘Just a tiny, tiny bit of DNA’^hh^

The second and related focal point is identity. One difficulty raised here is what is meant by identity, a topic that would readily take up several other volumes. Political, legal and ethical texts make various links between the concepts of identity and those of “genes” or “genomes”, and as Christine Hauskeller ( 2004 ) has argued these are often contradictory. With regard to mtDNA the current policy debates have constructed the issue as one of its (non-)contribution to identity, demonstrates:

The Appeal Committee accepted that Mitochondrial (sic) DNA is *not associated with identity or any pre-determined characteristics of the individual*. … [it] was satisfied that where pronuclear material is deposited into a new cell this does not change the genetic structure of the new cell because the nuclear material over-rides(sic) any DNA in the mitochondrial DNA ( HFEA 2005 ). (emphasis added)

Accordingly, the HFEA’s starting point was that mtDNA is not part of the genetic structure, and therefore is *not* identity determining. However, Lenny Moss has argued that there has been a conflation of two distinct historical types of concept, ‘gene P(henotype)’ and ‘gene D(NA)', which leads to the popular idea of having genes for characteristics ( Moss 2003 ). This conceptualisation is evident in the HFEA’s language above, which de-limits the role of mtDNA, but in doing so tends to suggest that there are indeed ‘genes for’ characteristics (eg eye colour^ii^ ), which does not recognise the complexity of genomics.

Subsequent policy documents have shown more nuanced approaches to the concept of identity. For example, the HGC focused on the ‘philosophically complex’ concept of ‘personal identity’, warning against ‘crude geneticism’ based on percentage contribution, and highlighting that ‘genetics is only one discourse’ relevant to an individual’s identity^jj^. The NCOB simply asked “How might mitochondrial DNA be associated with a person’s identity?” and raised questions as to the relationships created between the people involved in these new techniques^kk^; and in its subsequent Report considered four different notions of identity, namely ‘self-conception’, ‘qualitative’, ‘numerical’ and ‘genetic’ identity^ll^. Finally, more recently the HFEA has acknowledged the criticisms levelled at its stance in 2005^mm^, and reflected briefly on some philosophical approaches to personal identity (drawing on John Locke and John Searle)^nn^, before concluding that ‘the issue of personal identity is, and will probably remain, an unanswerable question.’ Limitations of space preclude further analysis of the rich discourse in these policy documents, which merit further consideration elsewhere.

Nonetheless, the HFEA also highlighted that ‘the majority of *scientific opinion* suggests that … [mtDNA] … would not affect a person’s essential characteristics (for example, a person’s hair or eye colour)’ (emphasis added)^oo^. A further example is provided by one of the key scientists and advisors to the HFEA, albeit in the context of rebutting the ‘three-parent’ headlines, thus: ‘This is not, however, “three-parent IVF”, said Professor Robin Lovell-Badge … “It is not a term we have used once in this report [HFEA Report, April 2011] and it is not a term that should be used,” he said. “This is a tiny, tiny bit of DNA. *It is not carrying any characteristics* except that you have normally functioning mitochondria”.’^pp^ (emphasis added) Hence, the rebuttal focused on the ‘size’ of the mtDNA, and once again on the types of characteristics. Whilst many *scientists* may be certain that a person’s ‘essential characteristics’ will be unaffected by mitochondrial donation, it is far from clear that the media will portray these developments in quite the same way^qq^, or what lay understandings will emerge. One reason this is significant is if/when these techniques are made lawful in the UK, in the future persons born following mitochondrial donation will have access to the media coverage. ‘Identity’ pertains not only to how we see ourselves, but how others view us. Hence, worries regarding *‘life meddling’ fears’* in the Daily Mail^rr^ are far from helpful, and also echo (potentially) damaging media coverage of the development of ARTs - from the (early) typification of sperm donors as ‘criminal’ to the fascination with Louise Brown’s development and procreative capacity.

The final issue considered here is the extent to which mtDNA is ‘identifiable’. The UK policy debates (outlined briefly above) have minimised the role which mtDNA can potentially play with regard to identification. Yet, mtDNA is a validated technique for the identification of skeletons in forensics ( Wilson et al. 1995 ; Holland et al. 1993 ); and also can be used in genealogy, with reference to ‘Mitochondrial Eve’^ss^. More controversially it has been used for ‘ethnicity testing’ for children with unknown parentage entering the care system ( Lucassen et al. 2010 ); and would have formed part of a proposed DNA based project to be piloted by the UK Border Agency, the ‘Human Provenance Pilot Project’, but this was dropped in March 2011. This project was reportedly driven by a desire to determine if the person was of the nationality which they claimed (from, for example, Somalia) or an economic migrant (from, for example, Kenya) – but the scientific community was damning as to the conflation of mtDNA for ancestry with that for race/nationality ( Brumfiel 2011 ; Balding et al. 2010 ). However questions remain as to whether different concepts of ‘identity’, and indeed alternative forms of state regulation (of ‘identity’) are used, depending on whether one is a refugee, a child in the care system, or conceived and born following mitochondrial donation.

### Practical matters

However, if for example mtDNA is constructed in policy as (potentially) significant to one’s identity, or if mitochondrial donors are to be treated akin to or in precisely the same way as other egg donors under the current regulatory framework, what implications might this have? Would there, for example, be a legal obligation to place details on the Register of Information with the HFEA^tt^ regarding the mitochondrial donation, and what type of information and to whom, if anyone, should it be made available to in the future? The Joint Committee scrutinising the Draft Bill noted that the situation was unclear:

*We suspect that the Government’s intention in this respect is that the child should have only two registered parents—those whose nuclear DNA was used to create the embryo—but that the child should be able to discover the identity of the female donor of mitochondrial DNA from the Register of Information in the same way as other donor-conceived individuals.* This is not entirely clear from the draft Bill and Explanatory Notes, although the Department of Health did provide further information on this point in a memorandum to the House of Lords Delegated Powers and Regulatory Reform Committee. This memorandum suggests that the power in clause 34 might, for example, be used to clarify that the woman who donated the egg with healthy mitochondria could not apply for a parental order on the basis that she *only contributed mitochondrial, not nuclear, DNA to the embryo*^uu^. (emphasis added).

Hence, the Joint Committee indicated that the mitochondrial donor was unlikely to be viewed as a legal parent on the basis of the provision of mtDNA alone; especially in light of the (expected) prohibition on such women seeking a parental order following surrogacy. However, the conditions determining eligibility for applying for parental orders do not require both intending parents to have a ‘genetic’ connection to the child in question, but instead specify only that the ‘gametes of at least one of the applicants’ were used^vv^ ( Biggs and Jones 2013 ). On a literal interpretation, therefore, the mitochondrial donor would have provided her own ‘gametes’, and any Regulations placed before Parliament for approval will need to be drafted clearly in order to delineate what exactly is meant by ‘gametes’ in this context.

The classification of the mitochondrial donor is also bound up in the broader regulatory context alluded to by the Joint Committee, including the Register of Information. To that end, Parliament inserted s.35A into the HFE Act 1990 to ensure that the pertinent provisions could be modified if mitochondrial donation is made lawful in the future. In addition to those governing parental orders, these provisions relate to the register of (donor) information (s.31 HFE Act 1990); the provision of information (s.31ZA-E), including requests by donor-conceived people as to their genetic parentage (s.31ZA); consents to the use or storage of gametes and embryos (Schedule 3); and the information to be provided to prospective parents under the licence conditions, regarding the early disclosure of the mode of their child’s (donor) conception (s.13(6C)). The stipulation of these factors does not, however, indicate that mitochondrial donors will necessarily be treated in the same way as egg donors. Rather, Parliament has simply ensured that the current regulatory framework has made provision for future clarification of their status, should it be necessary.

As mentioned above (under ‘parentage’), the question of how mitochondrial donors should be treated/classified has been raised by the HFEA and the NCOB. Both have asked whether the most suitable analogy (in regulatory terms) falls with organ/tissue donors or gamete donors; donors of other bodily materials (NCOB only^ww^), or a new ‘middle ground’ classification providing ‘a unique set of rights and responsibilities’^xx^. If mitochondrial donors are to be treated in the same way as ‘traditional’ egg donors then the regulatory position is clear ( Nuffield Council on Bioethics. 2012b ), whereas classification as tissue/organ donors would involve a demarcation between ‘egg’ donors and ‘mitochondrial’ donors. Nonetheless, there remains the possibility of a unique arrangement emerging.

The NCOB has recommended that ‘“motherhood” is not indicated either biologically or legally by virtue of mitochondrial donation’; accordingly the donor should *not* be accorded the same status as a traditional egg donor. Hence, although they were clear that the current fiscal arrangements and safety regulations for egg donors should also operate for mitochondrial donors, the ‘10 family’ limit (for gamete donations) was considered overly cautious. The NCOB further stated that mitochondrial donors should *not* be mandatorily identifiable in the future and that there was ‘no reason’ to establish sibling registries ( Nuffield Council on Bioethics. 2012b ). Interestingly therefore, immediately after these recommendations, the NCOB went on to suggest that a voluntary system *might* be ‘set up and mediated by an appropriate central body’, to facilitate contact between mitochondrial donors and offspring ( Nuffield Council on Bioethics. 2012b ). Yet no mention of such a system is made with regard to those sperm donors (solely) involved in the creation of embryos used in PNT cycles (ie whose pronuclear material will be discarded). Presumably this is due to the lack of *any* genetic tie with those born following these techniques. However, when viewed through a ‘collaborative reproduction’ lens, it is more difficult to demarcate with certainty who should/not be included on such a register, not least as there is a dearth of research on this issue ( Blyth et al. 2012 ).

## Conclusion

In this paper we have considered some of the ways in which mitochondrial donation has been framed in recent policy debates and consultation documents, with particular emphasis on ‘parentage’ and ‘identity’. One key consideration has been whether there is anything so novel about mitochondrial donation that it calls into question the current paradigms of understanding in Anglo-Welsh Law with regard to ‘parentage’ and the construction of donors in this context (defined broadly so as to include those providing gametes, organs, tissues, or other bodily materials). While we have concluded that mitochondrial donation is unlikely to call into question the current construction of legal parents (due to the primacy accorded to gestation), the classification of women donating mitochondria is likely to pose some problems for the policy-makers. Unlike most other types of donation, ie organ^yy^, tissue, blood and other bodily materials, mitochondrial donation not only alters the recipients’ genetic germline, but – for female offspring who go on to have their own children – this change is inter-generational. Hence, the direct analogies with the donation of tissue, blood, other bodily materials and organs (with the exception of ovaries), breaks down. It is unclear, therefore, how these egg/mitochondrial donors will be classified when Regulations are (as we assume they will be) eventually placed before Parliament for approval. Further, while the focus thus far in most of the media and policy debates concerns ‘serious’ debilitating ‘metabolic’ conditions^zz^, there is some evidence to link mitochondrial mutations with ageing and cancer( Kang et al. 2005 ; Chinnery 2002 ); so future research in this field may also yield information pertaining to broader health implications, which in turn may pose further regulatory conundrums.

## Endnotes

^a^Announced on 1 June 2012 (http://www.hfea.gov.uk/6896.html (accessed 1 June 2012)). See further http://mitochondria.hfea.gov.uk/mitochondria/ (accessed 02 November 2012). The consultation closed on 7 December 2012.

^b^M.S. Frankel and B.T. Hagen, Germline Therapies Background Paper, Nuffield Council on Bioethics Forward Look Seminar papers, 19–20 May 2011, accessible at (accessed 28 May, 2012): http://www.nuffieldbioethics.org/sites/default/files/files/Germline_therapies_background_paper.pdf; also, G. Watts, Mitochondria in the media, Bionews Comment, Bionews 661, 18 June 2012: http://www.bionews.org.uk/page_151704.asp (accessed 19 June 2012).

^c^See for example, the NCOB materials on mitochondria: http://www.nuffieldbioethics.org/mitochondrial-donation/mitochondrial-donation-background-what-are-mitochondria (accessed May 28, 2012).

^d^This account assumes a man and woman wishing to parent together; alternatively, for female same-sex couples and single women, it is assumed that a sperm donor would contribute half of the nDNA.

^e^http://www.hfea.gov.uk/6896.html (accessed 28 May 2012); NCOB op. cit. note 5.

^f^Due to the prohibitions imposed by s.3ZA HFE Act 1990, as amended; such embryos are not currently constructed in law as “permitted” embryos, see especially s.3ZA(4)(b).

^g^http://www.hfea.gov.uk/671.html (accessed 28 May 2012); see also ErikaCheck. UK embryo licence draws global attention. *Nature* 2005; 437: 315.

^h^Cited by Lord Walton in House of Lords, Hansard, 4 February 2008, Vol. 698(45), col 846–7. See also his earlier comments in the ‘Second Reading: First Sitting’ of the Human Fertilisation and Embryology Bill 2007–08 (HFE Bill) in the House of Lords, *Hansard*, 19 November 2007, Vol. 696(8), col 709; and Erika Check Hayden. A step towards three-parent babies? Progress report shows clinical application of technique still far away. *Nature News* 2008*;* doi:http://10.1038/news.2008.560.

^i^For example, by Lord Walton in his proposed amendment to the HFE Bill, Committee Stage: First Sitting: House of Lords, *Hansard*, 3 December 2007, Vol. 696(17), col 1504. For a brief discussion of a range of views see further: House of Lords and House of Commons. 2007. *Joint Committee on the Human Tissue and Embryos (Draft) Bill 2006–07 Volume 1: Report*: para 139–141, 182–186. In April 2011, Dr Evan Harris, writing for guardian.co.uk, urged the Government to act, ‘Our chance to stop mitochondrial diseases in their tracks’, http://www.guardian.co.uk/science/political-science/2011/apr/19/reproduction-embryos?INTCMP=SRCH (accessed 30 May 2012).

^j^Check Hayden, op.cit. note 15.

^k^3ZA(5) HFE Act 1990 (as amended) states that: “Regulations may provide that - (a) an egg can be a permitted egg, or (b) an embryo can be a permitted embryo, even though the egg or embryo has had applied to it in prescribed circumstances a prescribed process designed to prevent the transmission of serious mitochondrial disease”.

^l^This approach to regulation, using delegated legislation to avoid the need to return to Parliament for a full debate in both houses, was also used for the removal of anonymity for gamete donors in 2004, with the introduction and approval by Parliament of the Human Fertilisation and Embryology Authority (Disclosure of Information) Regulations, SI 2004 No 1511; for a discussion of the relevant debates in Parliament see C. Jones. 2007. *Why Donor Insemination Requires Developments in Family Law*. Lewiston, New York: Edwin Mellen Press, 231–242.

^m^HFEA, op. cit. note 3. Following its publication a joint open letter was sent to Andrew Lansley requesting the introduction of Regulations to parliament, signed by representatives of the Academy of Medical Sciences, Association of Medical Research Charities, Genetic Alliance UK, Medical Research Council, Muscular Dystrophy Campaign, Progress Educational Trust and the Wellcome Trust,April 2011http://www.wellcome.ac.uk/stellent/groups/corporatesite/@policy_communications/documents/web_document/wtvm051051.pdf (accessed 30 May 2012).

^n^See further A. Bainham. 1999. Parentage, Parenthood and Parental Responsibility, in *What is a Parent? A Socio-Legal Analysis*. A.Bainham, S.D. Sclater and M. Richards, eds. Oxford. Hart Publishing: 25–46, especially 28–30. Also, E. Jackson. 2006. What is a Parent? in *Feminist Perspectives on Family Law.* A. Diduck and K. O’Donovan, eds. Oxford: Routledge Cavendish GlassHouse, 59–74.

^o^Social constructions are considered in further detail under ‘identity’, below.

^p^House of Lords *Hansard*, December 3 2007, Vol. 696(17) Col 1502 and 1505 respectively.

^q^See further C. Jones. 2011. The (im)possible parents in law. In, *Taking Responsibility: Law and the Changing Family*. C. Lind, H. Keating, and J. Bridgeman, eds. Aldershot, GB, Ashgate, 201–220; and C. Jones. 2009. The identification of ‘parents’ and ‘siblings’: new possibilities under the reformed Human Fertilisation and Embryology Act. In, *Rights, Gender and Family Law*. J. Wallbank, A. Choudhry, and J. Herring, eds. Oxford, Routledge GlassHouse, 219–238; and A. Diduck. If only we can find the appropriate terms to use the issue will be solved: law, identity and parenthood. *Child and Family Law Quarterly* 2007; 19: 458–480.

^r^Check Hayden, op. cit. note 15.

^s^Briefing, *The Sunday Times*. 13 March 2011.

^t^D. Derbyshire. *Daily Mail*. 12 March 2011. http://www.dailymail.co.uk/health/article-1365287/Babies-THREE-parents-born-years-controversial-IVF-technique-gets-ahead.html (accessed 30 May 2012).

^u^Briefing, op. cit. note 30, emphasis added.

^v^See Prof. Lovell-Badge’s rebuttal of the phrase ‘three-parent IVF’ in Boseley, op. cit. note 32, discussed below.

^w^Frankel et al., op. cit. note 8: para 10; Check, op. cit. note 14; see also NCOB, op. cit. note 5: 2.13.

^x^For a brief discussion of some early outcomes of ooplasmic transfer see NCOB, op. cit. note 5: 2.10-2.12; Jacobs et al. The transmission of OXPHOS disease and methods to prevent this. *Human Reproduction Update* 2006; 12: 119–136, 129; Bredenoord et al. Ooplasmic and nuclear transfer to prevent mitochondrial DNA disorders: conceptual and normative issues. *Human Reproduction Update* 2008; 14: 669–678, 670.

^y^HGC. 2010. Discussion of ethical issues in human reproduction using material containing DNA from more than two sources. HGC 10/P07 – Annex A: para 25, available from http://www.hgc.gov.uk.

^z^HFEA. June 2011, ELAC (06/11) 1: 6.22, available from http://www.hfea.gov.uk/6896.html (accessed 30 May 2012).

^aa^Ibid. para 6.17.

^bb^NCOB, op. cit. note 4: 13.

^cc^This led the NCOB to question whether it would be appropriate to use PGD and sex-selection in order to select only male embryos for implantation, to avoid future potential problems for subsequent generations, see NCOB, op. cit. note 4: 11–14.

^dd^See s.33(1) HFE Act 2008: ‘The woman who is carrying or has carried a child as a result of the placing in her of an embryo or of sperm and eggs, and no other woman, is to be treated as the mother of the child.’ In addition, s.47 HFE Act 2008 makes clear that a woman cannot be considered to be the parent of a child simply by the fact of egg donation. Under the common law, see Lord Simon of Glaisdale’s statement that “[m]otherhood, although also a legal relationship, is based on a fact, being proved demonstrably by parturition”, in *The Ampthill Peerage Case* [1977] AC 547, 577.

^ee^Jones, 2011, op. cit. note 25: 210–212. See also Diduck, op. cit. note 25; McCandless, J. and Sheldon, S. The Human Fertilisation and Embryology Act (2008) and the Tenacity of the Sexual Family Form, *Modern Law Review* 2010;73: 175–207.

^ff^Law Commission, NZ (2005) *New Issues in Legal Parenthood* Report 88 Wellington: Law Comm.

^gg^*AA v BB & CC* (2007) ONCA 2. See also R. Leckey. The Practices of Lesbian Mothers and Quebec’s Reforms. *Canadian Journal of Women and the Law* 2011; 23: 579–99.

^hh^Boseley, op. cit. note 32, citing Prof. Robin Lovell-Badge.

^ii^See also, for example, BBC News, op.cit. note 27; also Derbyshire, op. cit. note 31 regarding an interview with a mother who had lost her son from a mitochondrial condition: ‘His eyes, face, hair would have been 100 per cent the same … the ultimate difference it would have made is that he would have survived.’ In contrast, HGA director Dr David King is also cited in the same article: ‘It is altering the genetic constituents in every cell in the child’s body. If it’s a girl, then those changes will be passed down to all her descendants’.

^jj^HGC, op. cit. note 2, para 22 – 37.

^kk^NCOB, op. cit. note 4: 10–11.

^ll^NCOB, op cit. note 5: 52–57.

^mm^HFEA ELAC, op. cit. note 39: 6.6.

^nn^Ibid, 6.8 – 6.17.

^oo^Ibid, 6.12.

^pp^Boseley, op. cit. note 32.

^qq^See for example, potential echoes of the GM crops reporting, where the focus was on tampering with nature, and questions as to whether changing one gene changed the nature of the plant; see further A. Hansen, Tampering with nature: ‘nature’ and the ‘natural’ in media coverage of genetics and biotechnology. *MCS* 2006; Vol. 28(6): 811–834.

^rr^Derbyshire, op. cit. note 28.

^ss^R. Tutton. “They want to know where they came from”: population genetics, identity, and family genealogy. *New Genetics and Society* 2004; Vol 23(1): 105–120. See also NCOB, op. cit. note 5 at 78–79 on ‘cultural representations of mitochondria and their inheritance’.

^tt^It has been suggested that the CQC will take over this aspect of the HFEA’s function, if and when the HFEA is disbanded (as indicated) under the Arm’s Length Bodies Review. See further the Department of Health. Consultation on proposals to transfer functions from the Human Fertilisation and Embryology Authority and the Human Tissue Authority. June 2012, http://webarchive.nationalarchives.gov.uk/20130107105354/http://www.dh.gov.uk/health/2012/06/consultation-regulators/ (accessed 02 November 2012).

^uu^Joint Committee, op. cit. note 16: para 185.

^vv^Section 54(1)(b) HFE Act 2008. See further C. Jones. 2011. op. cit. note 25 for a discussion of surrogacy and parenthood in Anglo-Welsh Law.

^ww^They do not define further what is meant by ‘other bodily material’ but see further NCOB. 2011. *Human Bodies: Donation for medicine and research*. Chapter 1.

^xx^HFEA, op. cit. note 39, 6.22-6.24; NCOB, op. cit. note 4: 11.

^yy^Ovary transplants are the exception here.

^zz^Including the statutory reference to ‘serious mitochondrial conditions’ per s.3ZA(5) HFE Act 1990.

## References

[CR1] Balding D (2010). Genetic and isotopic analysis and the UK Border Agency. RSS.

[CR2] BBC News (2008). Three-parent embryo formed in lab.

[CR3] Biggs H, Jones C, Cohen IG (2013). Tourism: a matter of life and death in the UK. The Globalization of Health Care.

[CR4] Blyth E, Crawshaw M, Frith L, Jones C (2012). Donor-conceived people’s views and experiences of their genetic origins: a critical analysis of the research evidence. JLM.

[CR5] Boseley S (2011). The Guardian.

[CR6] Brumfiel G (2011). UK immigration cancels DNA screening programme. Nature.com.

[CR7] Chinnery PF (2002). Accumulation of mitochondrial DNA mutations in ageing, cancer, and mitochondrial disease: is there a common mechanism?. The Lancet.

[CR8] Derbyshire D (2011). Daily Mail.

[CR9] Hamzelou J 2011.http://www.newscientist.com/blogs/shortsharpscience/2011/04/three-parent-babies-on-their-w.html

[CR10] Hauskeller C (2004). Genes, genomes and identity: projections on matter. New Genetics and Society.

[CR11] HFEA 2005.http://www.hfea.gov.uk./docs/R0153_How_the_decision_was_made_to_licence_this_research_project__2_.pdf

[CR12] HFEA (2011). Scientific review of the safety and efficacy of methods to avoid mitochondrial disease through assisted conception.

[CR13] HFEA (2012). Mitochondria Public Consultation 2012.

[CR14] Holland M (1993). Mitochondrial DNA sequence analysis of human skeletal remains: identification of remains from the Vietnam War. JFS.

[CR15] Human Genetics Commission (2010). Discussion of Ethical Issues in Human Reproduction Using Materials Containing DNA From More Than Two Sources.

[CR16] Kang D (2005). Alterations of mitochondrial DNA in common diseases and disease states: aging, neurodegeneration, heart failure, Diabetes and Cancer. Current Medicinal Chemistry.

[CR17] Lucassen A (2010). ‘Ethnicity testing’ before adoption: a help or hindrance?. Archives of Disease in Childhood.

[CR18] Moss L (2003). What Genes Can’t Do.

[CR19] Nuffield Council on Bioethics (2012). Emerging techniques to prevent inherited mitochondrial disorders: ethical issues.

[CR20] Nuffield Council on Bioethics (2012). Novel techniques for the prevention of mitochondrial DNA disorders: an ethical review.

[CR21] Robertson A (1999). Reconstituting eggs: The ethics of cytoplasm donation. Fertility and Sterility.

[CR22] Schwartz M, Vissing J (2002). Paternal inheritance of mitochondrial DNA. NEJM.

[CR23] Sciencewise-ERC: *the UK’s national centre for public dialogue in policy making involving science and technology issues*. http://www.sciencewise-erc.org.uk/ (accessed 28 May 2012)

[CR24] Wilson M (1995). Validation of mitochondrial DNA sequencing for forensic casework analysis. International Journal of Legal Medicine.

